# Cardiocerebral channelopathy caused by *KCND3* mutation in a child: A case report

**DOI:** 10.3389/fped.2022.1019122

**Published:** 2022-11-28

**Authors:** Yi Zhang, He Jiang, Xiao-mei Li

**Affiliations:** Department of Pediatric Cardiology, Heart Center, The First Hospital of Tsinghua University (Beijing Huaxin Hospital), Beijing, China

**Keywords:** early repolarization syndrome, epilepsy, cardiocerebral channelopathy, atrial fibrillation, quinidine

## Abstract

Early repolarization syndrome is rare in children. Mutation of genes encoding ion channels could display mixed electrophysiological phenotype of Kv4.3 including both cardiac phenotype (early repolarization syndrome, atrial fibrillation) and cerebral phenotype (epilepsy, intellectual disability). This situation is rare and was named as cardiocerebral channelopathy. Here, we report a case of an 11-year-old-girl with cardiocerebral channelopathy caused by *KCND3* mutation, who was successfully treated with oral quinidine, metoprolol and implantable cardioverter-defibrillator. Clinicians should be vigilant on the risk of cardiogenic syncope and sudden cardiac death in a patient with epilepsy, intellectual disability and early repolarization pattern.

## Introduction

Early repolarization pattern (ERP) is common in the general population and regarded as a benign electrocardiogram (ECG) phenomenon with an uneventful prognosis. Early repolarization syndrome (ERS) is diagnosed in a patient with an ERP on the ECG in addition to syncope or symptomatic arrhythmias, which significantly increase the risk of sudden cardiac death (SCD) (1, 2). However, to the best of our knowledge, ERS-related cardiocerebral channelopathy is extremely rare. Although the initial symptoms are epilepsy or intellectual disability, there is a possible SCD risk. Here, we report the case of an 11-year-old girl with ERS, atrial fibrillation (AF), epilepsy and intellectual disability caused by *KCND3* mutation and describe the patient's treatment and follow-up.

## Case presentation

**Figure 1:**
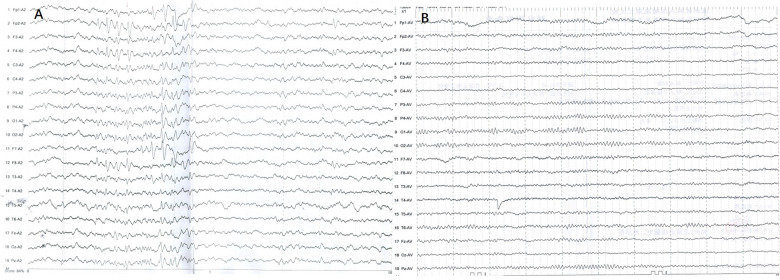
EEGs for this patients. (**A**) sharp waves and slow waves could be detected in each brain area which are predominant in anterior head lead. (**B**) EEG became normal during follow up.

**Figure 2:**
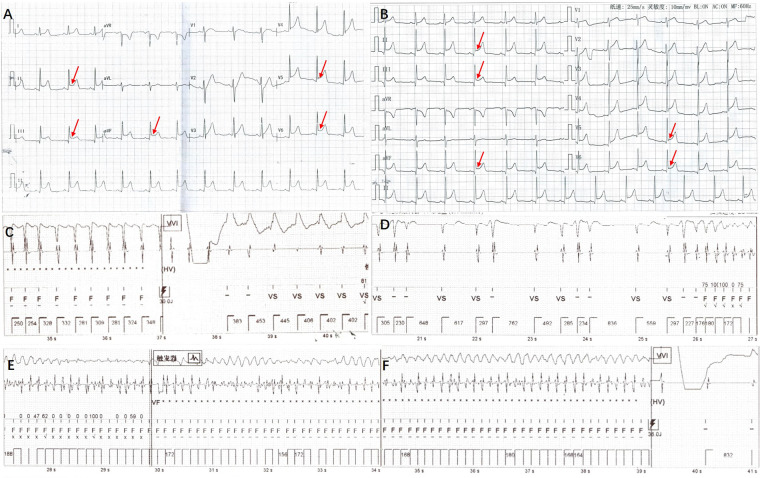
ECG and point diagram of ICD cavity for this patient. (**A**) ERP could be detected on ECG: elevation of J-point in inferior leads (II, III, AVF) and lateral leads (V5, V6). (**B**) J wave amplitudes were decreased slightly after administration of oral quinidine. (**C**) Diagram of ICD cavity shows atrial arrhythmias with electrical shock and ventricular pacing administered by ICD. (**D–F**) Diagram of ICD cavity shows VF occurred and was terminated by electrical shock of ICD.

An 11-year-old girl with a weight of 45.3 kg (P 75–90th) and a height of 148 cm (P 50–75th) was admitted to the Pediatric Cardiologic Department due to “nine-year recurrent loss of consciousness”. She was diagnosed with febrile seizures at the age of 2 and had regular febrile seizures from age four (seven years ago); the seizures occurred with loss of consciousness and eye gazing. The seizure would usually last for several seconds and then suddenly disappear. Electroencephalogram (EEG) showed bilateral temporal spikes, central spikes, slow spikes, multiple slow spikes, sharp waves and slow sharp waves, which were predominant in the left temporal area. Brain magnetic resonance imaging (MRI) results was normal. She was diagnosed with epilepsy and treated with oral sodium valproate. The patient's seizures were successfully controlled with antiepileptic medications. EEGs re-examined at six months showed sharp waves and slow waves in each brain area, especially in the anterior head lead (Figure 1A). Presently, she continues treatment with oral sodium valproate. Intellectual disability was diagnosed at six years old (Wechsler scale score < 75). ECG showed ERP (Figure 2A), and a 24 h Holter monitor showed paroxysmal AF, premature atrial complexes (PACs), paired PACs and paroxysmal atrial tachycardia (AT). The result of the echocardiogram was normal. She was admitted to the emergency room 2 years ago, at age 9, for 8 episodes of Adams-Stokes attack that occurred in one day. Cardiac monitoring showed ventricular fibrillation (VF). ERS was diagnosed, and oral metoprolol 25 mg bid (twice per day) was prescribed.

One year ago, at age 10, she woke and cried during sleep, presenting with recurrent Adams-Stokes attack, but was rescued successfully by cardiopulmonary resuscitation and defibrillation. A single chamber implantable cardioverter-defibrillator (ICD, CD1231-40Q, St. Jude Medical and lead, 7122Q/65, St. Jude Medical) was implanted. In addition to implantation of the ICD, she was treated successively by oral propranolol 10 mg tid (three times per day)→15 mg (morning) 15 mg (noon) 10 mg (evening), metoprolol 25 mg bid→50 mg tid and combined therapy of metoprolol 50 mg tid with verapamil 40 mg tid but the treatment was poorly efficacious. Repeated ECG and Holter monitor showed paroxysmal AT, paroxysmal AF and R-on-T premature ventricular complexes (PVCs). ICD recorded multiple episodes of inappropriate shocks during AT (ventricular rate 254–288 bpm) without syncope recurrence (Figure 2C). Three months before admission, she had a total of 10 episodes of Adams-Stokes attack over an 8-day period. A diagram of the ICD cavity showed VF during Adams-Stokes syndrome and successful defibrillation by ICD (Figures 2D–F). She was admitted for further treatment.

**Figure 3:**
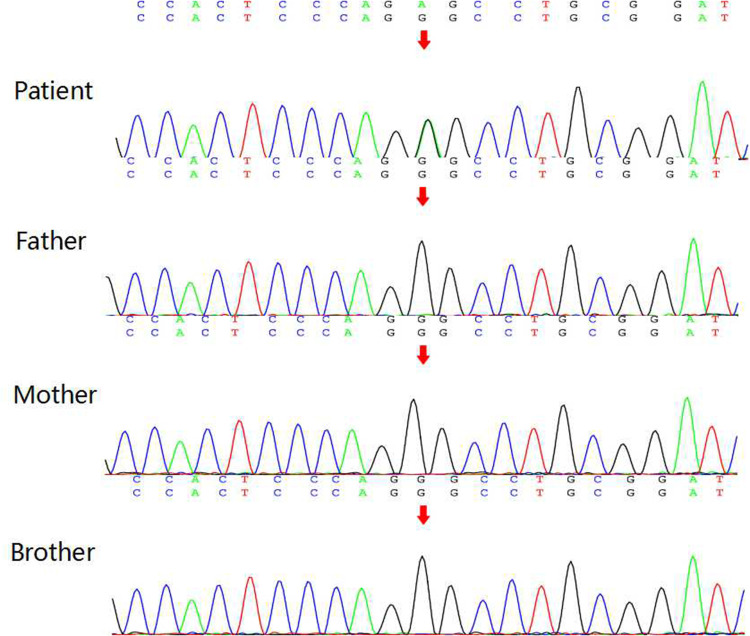
Results of *KCND3* gene DNA sequencing patient has *KCND3* chr 1-112524433 G306S (c.916G > A). Her parents and brother did not carry the same variant.

There was no family history of hereditary disease, syncope or sudden death. Whole-exome sequencing showed *KCND3* chr 1-112524433 and G306S (c.916G > A) (Figure 3, Beijing Maijinuo Medical Laboratory). The Sanger sequence was verified as a *de novo* mutation. According to ACMG guidelines, this variation evidenced PS2, PM2, PM5 [G306A had been reported (3)], PP3 (Bioinformatics protein function prediction software included SIFT, PolyPhen-2 and REVEL: harmful) and PP4 as a pathogenic mutation.

Diagnosis and treatment: After admission, oral metoprolol and verapamil were tapered gradually and ultimately withdrawn. Cardiac monitoring showed sinus rhythm with frequent short paroxysmal AT and occasional AF. Frequent R-on-T PVCs were detected. The diagnosis of ERS was clear, and the patient had a definite indication for ICD implantation. Because of atrial and ventricular arrhythmia, the patient suffered inappropriate shocks and electrical storms, for which antiarrhythmic drug therapy is appropriate. Because of the definitive role of the pathogenic gene and ERS diagnosis, propranolol, metoprolol and verapamil were ineffective, and we decided to administer quinidine, which was administered orally at 50 mg bid and gradually increased to 66.7 mg bid. Repeated ECGs showed no QT interval prolongation. PVCs disappeared after the administration of oral quinidine. Lasting several minutes up to one hour, paroxysmal short AT and paroxysmal AF with a ventricular rate of 110–130 bpm could still be detected 10 days after adding oral quinidine and usually occurred when the girl became angry. Atrial arrhythmias disappeared after a low dose of oral metoprolol 12.5 mg bid was added. Holter monitoring showed sinus rhythm with a total of 117,185 beats per 24 h. During 12 months of follow-up, no ICD shocks were detected. Holter monitor tests on three occasions showed no atrial or ventricular arrhythmias. ECG showed that the J wave amplitude decreased slightly (Figure 2B). There were no epileptic seizures, and her EEG result was normal (Figure 1B). Under the guidance of a pediatric neurologist, the sodium valproate dose is tapered. Antiarrhythmic drug therapy and evaluation of therapeutic effect are summarised in Table 1.

**Table 1:** Summary of antiarrhythmic drug therapy and evaluation of therapeutic effect.

	Age	Drug	Dose	AT or AF (bpm)	VF	Syncope	ICD shocks	
								VF	Inappropriate
Before admission	9y4m-10y	Metoprolol	50 mg/day	+	210	+	+		Implant
	10y1m	Propranolol	30–40 mg/day	+	173–222	+	+	+	+
	10y2m-10y10m	Metoprolol	50 mg/day	+	170–240	+	+	+	+
	10y11m-11y3m	Metoprolol	75–150 mg/day	+	173–204	+	+	+	+
	11y4m-11y6m	Metoprolol + Verapamil	150 mg/d + 120 mg/day	+	130–193	+	+	+	−
After admission	11y6m-12y	Quinidine	100–133 mg/day	+	110–130	−	−	−	−
		Quinidine + Metoprolol	133 mg/day + 25 mg/day	−	−	−	−	−	−

AT, atrial tachycardia; AF, atrial fibrillation; bpm, beat per minute; VF, ventricular fibrillation; ICD, implantable cardioverter-defibrillator; +, positive; −, negative.

## Discussion

*KCND3* encodes Kv4.3 protein, ɑ subunit of the fast transient outward potassium (Ito) channel, which expressed on both cardiac and cerebral tissues. Clinical manifestations include both cardiac phenotype (ERS, AF) and cerebral phenotype (epilepsy, intellectual disability), collectively named cardiocerebral channelopathy (4). Gain of function of the potassium ion channel caused by the *KCND3* mutation can lead to Brugada syndrome, early-onset AF and ERS (3), while loss of function of the potassium ion channel from the *KCND3* mutation can lead to spinocerebellar ataxia (5). Recently, four cases(all of them over 12 years of age) were reported with *KCND3* G306A or V392I mutations displaying a mixed electrophysiological phenotype of Kv4.3, including both cardiac and cerebral phenotypes (3, 6). The pathogenesis of the cardiac phenotype can be explained by the increased fast transient outward potassium current (3). Genetic analysis of this patient identified a *KCND3* G306S mutation similar to the *KCND3* G306A mutation reported in the literature. Both mutations affect the amino acid at position 306, which is a transmembrane region, and have similar phenotypes, suggesting that this mutation led to cardiocerebral channelopathy in our patient.

ERP has been regarded as a benign ECG phenomenon with an excellent prognosis (7). However, many recent studies have shown that ERP significantly increases the risk of SCD, cardiac death and death from any cause (2). There are no convincing studies that identify the epidemiological features of ERP in children and ERS is rare in children. Inferolateral with anterior ERP has been a key predictor for poor prognosis (8). The initial symptoms of our case were seizures, and ERP was discovered accidentally: inferior and lateral ERP on her ECG, which are concordant with the predictors of a poor prognosis as reported in the literature. Multiple occurrences of VF and surviving cardiac arrest after cardiopulmonary resuscitation indicate a high risk of SCD. Clinicians should be vigilant for cardiocerebral channelopathy and the risk of cardiogenic syncope and SCD in patients with epilepsy, intellectual disability and ERP.

It has been reported that at least 40% of ERS patients with a history of SCD or VF had VF recurrence during follow-up; therefore, implantation of ICD is recommended for patients who show ERP on their ECGs and also have a history of cardiac arrest or persistent ventricular arrhythmias (1, 9). A study showed that in patients with idiopathic VF and ICD implantation, inappropriate shock caused by AF was common (40%) (9). Our patient is a survivor of cardiac arrest with definite indication of ICD implantation and had atrial arrhythmias (paroxysmal AF and AT) in addition to ERS. Since a fast ventricular rate of atrial arrhythmias may lead to inappropriate shock by ICD, antiarrhythmic medication is indicated to control the atrial arrhythmias. Previous studies have shown that isoproterenol can effectively suppress VF electrical storms in patients with ERS and that the long-term use of quinidine can prevent the recurrence of ventricular arrhythmias (10, 11). Other antiarrhythmic medications have very limited effects in the prevention of ventricular arrhythmia recurrence (10). In our case, ventricular arrhythmias disappeared after quinidine treatment. The mechanism might quinidine could normalize the gain-of-function changes of mutant Kv4.3 (3). This was a case of ion channel disease caused by gene mutation, and the patient might present with ventricular arrhythmia and suffer the risk of syncope and SCD. Oral quinidine is safe and effective for ventricular arrhythmia, but long-term or lifelong quinidine therapy is often required. It should be emphasised that the Kv4.3 current increases during bradycardia because more Kv4.3 channels can recover from inactivation. Bradycardia might, possibly, worsen symptoms (12). In this case, low-dose oral metoprolol was added to control the atrial arrhythmias without causing bradycardia, and these anti-arrhythmic drugs did not increase the risk of ventricular arrhythmia. We still need follow-up to focus on bradycardia and ventricular arrhythmias.

## Conclusions

Cardiocerebral channelopathy should be suspected in a patient with epilepsy and intellectual disability whose ECG shows ERP and AF. Quinidine is safe and effective for children with ERS.

## Strengths and limitations

This was a very rare case. Since the *KCND3* mutation present as a mixed electrophysiological phenotype of Kv4.3, including both cardiac and cerebral phenotype, doctors should be vigilant on the risk of cardiogenic syncope and SCD. Limitations: This case did not permit further functional study, but would be considered if future conditions permit.

## Data Availability

The original contributions presented in the study are included in the article/Supplementary Material, further inquiries can be directed to the corresponding author/s.
